# Setting the stage for a role of the postsynaptic proteome in inherited neurometabolic disorders

**DOI:** 10.1007/s10545-018-0240-x

**Published:** 2018-08-21

**Authors:** Àlex Bayés

**Affiliations:** 1 Molecular Physiology of the Synapse Laboratory, Biomedical Research Institute Sant Pau (IIB Sant Pau), C/Sant Antoni M. Claret, 167, 08025 Barcelona, Spain; 2 Universitat Autònoma de Barcelona, Bellaterra, Cerdanyola del Vallès Spain

## Abstract

Neurotransmitter diseases are a well-defined group of metabolic conditions caused, in most instances, by genes specifically expressed in the presynaptic button. Better understanding of presynaptic molecular physiology, both in normal and pathological conditions, should help develop therapeutical strategies. The clinical relevance of the presynapse in inherited metabolic disorders is in glaring contrast with that of the postsynaptic component, which so far does not seem to play a relevant role in these disorders. This is somewhat surprising, as postsynaptic proteins are known to be involved in many nervous system diseases, particularly in neurodevelopmental and psychiatric disorders. The goal of this article is to explore if defects in the sophisticated postsynaptic machinery could also have a role in neurometabolic disorders.

**Electronic supplementary material:**

The online version of this article (10.1007/s10545-018-0240-x) contains supplementary material, which is available to authorized users.

## Introduction

Synapses are pivotal in cognition and behaviour, as they decode and store sensorial information. For this reason, they are very often involved in the pathophysiology of mental disorders. Synapses are typically divided in three compartments: the presynaptic button, the synaptic cleft and the postsynaptic element. The postsynaptic element of glutamatergic synapses, which represent the vast majority of central nervous system synapses (Beaulieu and Colonnier 1985; von Bohlen Und Halbach 2009; Defelipe et al. 2002), has a unique morphology, which is essential to its function. This structure was first described by Santiago Ramón y Cajal who named it ‘dendritic spine’ (Yuste 2015). Dendritic spines are dynamic protrusions of the postsynaptic membrane that present a bulbous head connected to the dendritic shaft through a thin neck (von Bohlen Und Halbach 2009). Another characteristic feature of dendritic spines is the presence of a very large protein complex beneath the postsynaptic membrane, the postsynaptic density (PSD). Recent large-scale proteomics experiments have produced a very detailed catalogue of the proteins which are present at the PSD, identifying hundreds of different proteins (Bayés and Grant 2009; Bayés et al. 2012, 2017; Distler et al. 2014; Focking et al. 2016; Roy et al. 2018). Paramount among PSD proteins are neurotransmitter receptors, which mediate the propagation of the incoming action potentials. Nevertheless, the PSD contains many other protein types, which participate in the translation of the electrical input into chemical signals, which ultimately drive the functional state of the synapse (Boeckers 2006; Kim and Sheng 2009; Sheng and Kim 2011; Dosemeci et al. 2016).

Neurotransmitter disorders are a group of inherited neurometabolic syndromes that are primarily caused by an altered bioavailability of neurotransmitter at the synapse (Pearl et al. 2007; Hoffman and Blau 2014; Marecos et al. 2014; Ng et al. 2015; Cortès-Saladelafont et al. 2016). They are generally caused by pathogenic mutations in genes expressed at the axon terminal. These code for enzymes involved in the synthesis or degradation of neurotransmitters. Nevertheless, genes coding for proteins responsible for neurotransmitter release and reuptake have also been reported mutated in these conditions. The presynaptic space has thus an important role in neurometabolic disorders. Pathophysiological descriptions of neuronal dysfunction in many other classic inborn errors of metabolism (intoxication disorders, energy defects and complex molecule defects) have been mainly described from a presynaptic perspective. This circumstance is in strong contrast with our understanding of the postsynaptic role in neurometabolic disorders. In this article, I explore what is known about the main metabolic pathways functioning at the postsynapse and their potential relevance in the field of neurometabolic disorders.

## Postsynaptic metabolic pathways

**Fig. 1:**
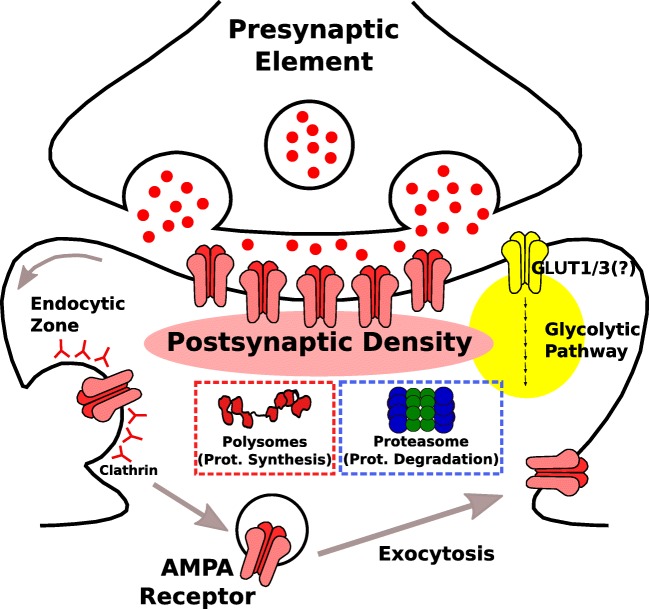
Schematic representation of a glutamatergic synapse indicating major metabolic pathways identified in the postsynaptic proteome. Proteomic evidence suggests that the glucose transporters GLUT1 and GLUT3  as well as the entire glycolytic pathway (represented as a yellow circle) would be present at the postsynaptic site. Confirmation of this observation with alternative methodological approaches would be required to confidently locate the glycolytic pathway within the PSD. Polysomes, for protein synthesis, and the proteasome, for protein degradation, are shown to represent the enrichment in the PSD of pathways related to protein metabolism. Finally, the traffic of AMPA receptors in and out of the postsynaptic membrane is also shown, indicating the endocytic zones where clathrin-mediated endocytosis of AMPA receptors occurs

A close inspection to the postsynaptic proteome should readily inform us about the key metabolic pathways operating in it. To achieve this purpose, I have looked for molecular pathways in a reference PSD proteome (Bayés et al. 2017) using the information contained in the ‘Reactome Pathways Database’ (Fabregat et al. 2018). The bioinformatics analysis tools provided by ‘Panther Classification System’ (Mi et al. 2016) have been used to identify metabolic pathways significantly enriched in the PSD, as previously reported (Bayés et al. 2012; Reig-Viader et al. 2018). If a pathway is significantly enriched in the PSD, it means that it presents a higher number of components than would be expected by chance. The vast majority of pathways that have been identified in this exercise are intracellular signal transduction pathways (see Supplementary Table 1), which is in accordance with our current knowledge of the molecular characteristics of the postsynaptic proteome (Boeckers 2006; Kim and Sheng 2009; Sheng and Kim 2011; Bayés et al. 2012; Dosemeci et al. 2016). These pathways typically involve the activation of membrane receptors, G-proteins or small GTPases that result in a series of molecular events, mostly phosphorylation cascades, which ultimately promote changes in cellular physiology. Nevertheless, a number of canonical metabolic pathways could be identified in the postsynaptic machinery. These are primarily related to the energetic and protein metabolisms (Table 1). However, if one considers the expanded definition of inborn errors of metabolism (IEM), in which dysfunctions of protein traffic are also regarded as metabolic conditions (García-Cazorla and Saudubray, this issue), the list of postsynaptic metabolic pathways is extended (Table 1 and Fig. 1). Interestingly, this analysis also revealed that while the presynaptic button presents a set of specific metabolic pathways, the postsynaptic element does not seem to contain a similarly unique metabolism. With the exception of a pathway involved in the trafficking of α-amino-3-hydroxy-5-methyl-4-isoazolepropionic (AMPA) glutamate receptors, postsynaptic metabolic pathways underlay basic cellular functions. This is relevant, as it means that proteins involved in these processes are not unique to the postsynapse and thus a direct link between a mutation and a postsynaptic role in disease would not be straightforward.

**Table 1:** Postsynaptic metabolic pathways

Pathway name	Pathway code	Postsynaptic proteins in pathway
Energetic metabolism		
Glycolysis	R-MMU-70171	9
Translocation of GLUT4 to the plasma membrane	R-MMU-1445148	38
Protein metabolism		
Protein translation	R-MMU-72766	62
Chaperonin-mediated protein folding	R-MMU-390466	22
Different proteasome pathways	R-MMU-5610785; R-MMU-195253; R-MMU-5610780	33
Endocytosis and traffic of neurotransmitter receptors		
Trafficking of AMPA receptors	R-MMU-399719	25
Clathrin-mediated endocytosis	R-MMU-8856828	43

### Energetic metabolism at the postsynapse

The presence at the presynaptic button of glucose transporters, glycolytic enzymes and mitochondria is well documented (Jang et al. 2016; Ashrafi et al. 2017). These importantly contribute to the energetic demands posed by presynaptic activity (Harris et al. 2012; Rangaraju et al. 2014). On the other hand, our understanding of the energetic machinery present at the postsynaptic side is by no means well established. The existence of glucose transporters and glycolytic enzymes in the postsynapse is still controversial. Furthermore, mitochondria do not have access to dendritic spines, remaining in dendritic shafts (Li et al. 2004; von Bohlen Und Halbach 2009). Actually, mitochondrial ATP is thought to freely diffuse into the spine head (von Bohlen Und Halbach 2009), instead of being actively transported there. This is particularly puzzling if we consider that most brain energy is consumed by synapses, and within synapses, restoring the postsynaptic membrane potential alone requires 50% of all its energetic demand (Attwell and Laughlin 2001; Harris et al. 2012).

Several PSD proteomics experiments have identified glucose transporters 1 (GLUT1) and 3 (GLUT3), the former being more frequently reported (Fernandez et al. 2009; Bayés et al. 2011, 2012, 2017; Distler et al. 2014; Focking et al. 2016). Other glucose transporters are normally not found in proteomic analyses of postsynaptic preparations. Nevertheless, these studies commonly find GLUT1 and GLUT3 in low amounts, so that, without evidence from other methodological approaches, their location at the postsynapse remains somewhat uncertain. The literature on brain cells expressing GLUT1 is neither fully conclusive. While there is evidence that GLUT1 is expressed in blood vessels and astrocytes, its expression in neurons cannot be discarded (Leino et al. 1997; Simpson et al. 2007; Jurcovicova 2014). Instead, the expression of GLUT3 in neurons is well documented (Leino et al. 1997; Simpson et al. 2007; Jurcovicova 2014). The presence of GLUT3 at synapses has been confirmed by an immunofluorescence co-localisation experiment, although its precise sub-synaptic location (pre- vs. postsynaptic) could not be fully established (Ferreira et al. 2011).

Proteomics experiments also report glycolytic enzymes at the PSD (Fernandez et al. 2009; Bayés et al. 2011, 2012, 2017; Distler et al. 2014; Focking et al. 2016). Actually, these articles usually identify the complete set of glycolytic enzymes. Nevertheless, to the best of my knowledge, the location at the PSD of glycolytic enzymes has only been proven once using other experimental approaches (Wu et al. 1997). In this seminal article, glyceraldehyde-3-phosphate dehydrogenase (GAPDH) was localised to the PSD by electron microscopy using specific antibodies. Furthermore, the activity of phosphoglycerate kinase (PGK), GAPDH and lactate dehydrogenase (LDH) was measured in biochemical preparations of postsynaptic densities proving that ATP can be synthesised at the PSD. This pioneer work suggests that some of the ATP consumed at the PSD would be locally produced via glycolysis, instead of coming from dendritic mitochondria. They also suggest that some postsynaptic functions require an instant and spatially controlled supply of ATP. As it occurs in synaptic vesicles, which contain both GAPDH and PGK that produce ATP in a highly localised manner for efficient neurotransmitter loading (Ikemoto et al. 2003). Further research will be required to completely clarify if glycolytic enzymes play a relevant function at the PSD, and if their malfunction could have a role in disease. Besides glycolysis, the pathway ‘Translocation of GLUT4 to the plasma membrane’ has also been found enriched among PSD proteins (Table 1). Out of the 67 proteins that constitute this pathway, 38 are at the PSD, representing a sixfold enrichment from what would be expected by chance (Fisher’s exact test *p* = 9.5 E-14, Supplementary Table 1). Nonetheless, it is important to remark that GLUT4 is not found in the postsynaptic proteome, neither has it ever been localised to dendritic spines using alternative experimental approaches. Thus, these 38 proteins might be involved in the traffic of other glucose transporters.

Finally, the postsynaptic proteome presents many proteins involved in the Krebs cycle and oxidative phosphorylation (see Supplementary Table 1), two metabolic processes that occur within mitochondria although, as has been already mentioned, these are absent from the postsynaptic compartment. Nevertheless, the presence of contaminating mitochondria in biochemical preparations of the PSD is as well documented as difficult to avoid (Carlin et al. 1980). Mitochondrial proteins, and the pathways they are involved in, are not considered as true components of the PSD.

### Protein metabolism at the postsynapse

Protein synthesis is required for the long-term changes in synaptic plasticity that underpin the formation of long-lasting memories (De Robertis and Bennett 1954; Palade and Palay 1954; Gray 1959; Martin et al. 2000). One of the first indications that proteins are synthesised outside the cell soma was actually obtained from the observation of polysomes at neuronal dendrites, close to postsynaptic spines (Steward and Levy 1982; Spacek 1985). Later, polysomes were shown to selectively enter dendritic spines that had been stimulated to produce a long-term potentiation (Ostroff et al. 2002). On the bases of all these findings, it should not come as a surprise that several molecular pathways related to protein metabolism appear as enriched at the postsynapse (Table 1 and Fig. 1). These include pathways involved in protein translation but also pathways important for protein turnover, such as ‘Chaperonin-mediated protein folding’ and protein degradation via the proteasome. The control of protein degradation by the ubiquitin proteasome pathway is tightly regulated at postsynaptic dendritic spines (Ehlers 2003). As it occurs for polysomes, the proteasome complex also displays a dynamic localisation between dendritic shafts and spines, which is under the control of synaptic activity (Bingol and Schuman 2006).

### Traffic of AMPA glutamate receptors and postsynaptic endocytosis

AMPA glutamate receptors (AMPAR) are the main drivers of fast excitatory neurotransmission. As a general rule, an increase in AMPAR at the synapse results in synaptic potentiation, while the opposite results in synaptic depression (Shepherd and Huganir 2007). Thus, their traffic in and out of the postsynaptic membrane is tightly regulated (Anggono and Huganir 2012). The insertion of AMPARs in the postsynaptic membrane occurs via exocytosis of cytosolic vesicles containing these receptors and later lateral diffusion towards the PSD. Similarly, endocytosis is required to remove AMPAR from the synapse. The areas of endocytosis at dendritic spines have been termed endocytic zones (Racz et al. 2004); these promote clathrin-mediated endocytosis of AMPARs and other cargo leaving the synapse. Proteins involved in AMPAR traffic and clathrin-mediated endocytosis are involved in the last two metabolic pathways characteristic of the postsynaptic machinery (Table 1). Because of the specialised machinery involved in AMPAR traffic, this pathway is likely to be the only metabolic pathway really specific to the postsynaptic proteome.

## Postsynaptic metabolic disorders

As previously introduced, neurometabolic disorders are intimately associated with presynaptic physiology. The notion of postsynaptic metabolic disorders is, at this point, rather speculative, with hardly any scientific literature supporting it. It is plausible that neurotransmitter diseases will secondarily alter postsynaptic physiology. Actually, this has already been reported for the creatine transporter deficiency, which results in increased synaptic levels of creatine and a prolonged stimulation of GABA receptors, to which the postsynaptic neuron responds by reducing the number of inhibitory synapses (Salomons et al. 2003). Similar pathophysiological mechanisms have been shown for the SSADHD deficiency (Pearl et al. 2009; Reis et al. 2012). Nevertheless, in this section, I wish to explore if there is evidence suggesting that primary dysfunction of the postsynaptic metabolic pathways described above could be involved in neurological conditions. To achieve this goal, we have looked for those genes coding for proteins involved in postsynaptic metabolic pathways that when mutated cause inherited brain conditions. It is important to keep in mind that most postsynaptic metabolic pathways are not exclusive to the postsynapse. Thus, their mutation will not necessarily imply a clinically relevant affectation of the postsynaptic physiology.

### Fifty-three PSD proteins involved in metabolic pathways cause inherited disease

The ‘Online Mendelian Inheritance in Man’ (OMIM) database (McKusick 2007) was first used to identify proteins from postsynaptic metabolic pathways causing disease. I later gathered the individual symptoms that constitute these conditions from the ‘Human Phenotype Ontology’ (HPO) database (Köhler et al. 2017), which systematically brakes down clinical conditions into their main symptoms (or phenotypes). According to OMIM, of the 232 PSD proteins involved in postsynaptic metabolic pathways, 53 cause inherited clinical disorders. Table 2 lists these proteins with the name of the disease they are involved in. As some genes cause more than one condition, the total number of medical conditions caused by these 53 genes raises to 60.

**Table 2:** Proteins involved in postsynaptic metabolic pathways that cause inherited diseases

Pathway	Gene name	Disease (OMIM)	OMIM ID
Energetic metabolism			
Glycolysis (including glucose transporters)			
	*ALDOA*	GLYCOGEN STORAGE DISEASE XII	611881
	*HK1*	NEUROPATHY, HEREDITARY MOTOR AND SENSORY, RUSSE TYPE	605285
	*PFKM*	GLYCOGEN STORAGE DISEASE VII	232800
	*SLC2A1*	GLUT1 DEFICIENCY SYNDROME 2	612126
	*TPI1*	TRIOSEPHOSPHATE ISOMERASE DEFICIENCY	615512
Translocation of GLUT4 to the plasma membrane			
	*MYH9* (1)	SEBASTIAN SYNDROME	605249
	*MYO5A*	GRISCELLI SYNDROME, TYPE 1	214450
	*TUBA4A*	AMYOTROPHIC LATERAL SCLEROSIS 22	616208
	*TUBA8*	CORTICAL DYSPLASIA, COMPLEX, WITH OTHER BRAIN MALFORMATIONS 8	613180
	*TUBB2A*	CORTICAL DYSPLASIA, COMPLEX, WITH OTHER BRAIN MALFORMATIONS 5	615763
	*TUBB2B*	CORTICAL DYSPLASIA, COMPLEX, WITH OTHER BRAIN MALFORMATIONS 7	610031
	*TUBB3*	FIBROSIS OF EXTRAOCULAR MUSCLES, CONGENITAL, 3A	600638
	*YWHAE*	MILLER-DIEKER LISSENCEPHALY SYNDROME	247200
	*YWHAG*	EPILEPTIC ENCEPHALOPATHY, EARLY INFANTILE, 56	617665
Protein metabolism			
Protein translation			
	*RPL21*	HYPOTRICHOSIS 12	615885
	*RPL26*	DIAMOND-BLACKFAN ANEMIA 11	614900
	*RPS10*	DIAMOND-BLACKFAN ANEMIA 9	613308
	*RPS19*	DIAMOND-BLACKFAN ANEMIA 1	105650
	*RPS28*	DIAMOND-BLACKFAN ANEMIA 15	606164
	*RPS29*	DIAMOND-BLACKFAN ANEMIA 13	615909
	*RPS14*	CHROMOSOME 5q DELETION SYNDROME	153550
	*RPL11*	DIAMOND-BLACKFAN ANEMIA 7	612562
	*RPL35A*	DIAMOND-BLACKFAN ANEMIA 5	612528
	*RPS23*	BRACHYCEPHALY, TRICHOMEGALY, AND DEVELOPMENTAL DELAY	617412
	*RPL5*	DIAMOND-BLACKFAN ANEMIA 6	612561
Chaperonin-mediated protein folding			
	*CCT5*	NEUROPATHY, HEREDITARY SENSORY, WITH SPASTIC PARAPLEGIA	256840
	*CSNK2A1*	OKUR-CHUNG NEURODEVELOPMENTAL SYNDROME	617062
	*GNAI2*	VENTRICULAR TACHYCARDIA, FAMILIAL	192605
	*GNAI3*	AURICULOCONDYLAR SYNDROME 1	602483
	*GNAO1*	NEURODEVELOPMENTAL DISORDER WITH INVOLUNTARY MOVEMENTS	617493
	*GNAO1*	EPILEPTIC ENCEPHALOPATHY, EARLY INFANTILE, 17	615473
	*GNB3*	NIGHT BLINDNESS, CONGENITAL STATIONARY, TYPE 1H	617024
	*GNB4*	CHARCOT-MARIE-TOOTH DISEASE	615185
	*GNB5*	LANGUAGE DELAY AND ATTENTION DEFICIT-HYPERACTIVITY DISORDER	617182
	*RGS9*	PROLONGED ELECTRORETINAL RESPONSE SUPPRESSION	608415
Different proteasome pathways			
	*APC*	FAMILIAL ADENOMATOUS POLYPOSIS 1	175100
	*CTNNB1*	MENTAL RETARDATION, AUTOSOMAL DOMINANT 19	615075
	*PPP2R1A*	MENTAL RETARDATION, AUTOSOMAL DOMINANT 36	616362
Endocytosis and traffic of neurotransmitter receptors			
Trafficking of AMPA receptors			
	*AP2S1* (2)	HYPOCALCIURIC HYPERCALCEMIA, FAMILIAL, TYPE III	600740
	*CACNG2*	MENTAL RETARDATION, AUTOSOMAL DOMINANT 10	614256
	*CAMK2A*	MENTAL RETARDATION, AUTOSOMAL DOMINANT 53	617798
	*CAMK2B*	MENTAL RETARDATION, AUTOSOMAL DOMINANT 54	617799
	*EPB4.1 L1*	MENTAL RETARDATION, AUTOSOMAL DOMINANT 11	614257
	*GRIA3*	MENTAL RETARDATION, X-LINKED, SYNDROMIC, WU TYPE	300699
	*GRIA4*	NEURODEVELOPMENTAL DISORDER WITH OR WITHOUT SEIZURES AND GAIT ABNORMALITIES	617864
	*GRIP1*	FRASER SYNDROME 3	617667
	*MYO6*	DEAFNESS, AUTOSOMAL DOMINANT 22	606346
	*PRKCG*	RETINITIS PIGMENTOSA 11	600138
	*PRKCG*	SPINOCEREBELLAR ATAXIA 14	605361
Clathrin-mediated endocytosis			
	*ACTB* (3)	BARAITSER-WINTER SYNDROME 1	243310
	*ACTG1* (3)	BARAITSER-WINTER SYNDROME 2	614583
	*DNM2*	CHARCOT-MARIE-TOOTH DISEASE	606482
	*NECAP1*	EPILEPTIC ENCEPHALOPATHY, EARLY INFANTILE, 21	615833
	*PIP5K1C*	LETHAL CONGENITAL CONTRACTURE SYNDROME 3	611369
	*WNT5A*	ROBINOW SYNDROME, AUTOSOMAL DOMINANT 1	189700

(1) This gene causes six different syndromes with similar phenotypes, none of which are of a clear neurological nature

(2) AP2S1 is also in the group of proteins implicated in ‘Clathrin-mediated endocytosis’

(3) ACTB and ACTG1 are also in the group of proteins implicated in ‘Translocation of GLUT4 to the plasma membrane’

### Most inherited diseases caused by metabolism proteins in the PSD show neurological symptoms

The phenotypic information obtained from HPO allowed us to identify those clinical conditions from OMIM that present neurological manifestations. Interestingly, of all 60 disorders, only eight did not cause neurological symptoms (Supplementary Table 2 and Table 3). This indicates that the majority of these proteins perform important functions in the brain and may be also at the postsynaptic level. The list of identified disorders includes (i) eight forms of intellectual disabilities, (ii) three epileptic encephalopathies, (iii) three neurodevelopmental conditions, (iv) three types of cortical dysplasia and (v) one lissencephaly, as well as neuropathies such as Charcot-Marie-Tooth diseases (Supplementary Table 2). When looking at disease types caused by proteins involved in the same metabolic pathways, we see that (i) large brain malformations such as cortical dysplasias and lissencephaly are caused by proteins—mostly tubulins—involved in the pathway ‘Translocation of GLUT4 to the plasma membrane’ and (ii) intellectual disabilities are very common among proteins involved in ‘Trafficking of AMPA receptors’, which is likely the pathway most specific to postsynaptic function. Finally, I have identified many anemias caused by proteins involved in protein translation, although these present few neurological symptoms, besides migraine (see Supplementary Table 2).

**Table 3:** Number of diseases presenting major neurological symptoms

Pathway	Intellectual disability	Seizures	Microcephaly	Generalised hypotonia	Migraine	Specific learning disability	Hypoplasia of the corpus callosum	Hyporeflexia	Spasticity
Energetic metabolism									
Glycolysis (including glucose transporters)	2	2	1	1	0	1	0	2	3
Translocation of GLUT4 to the plasma membrane	2	7	3	2	0	2	4	1	2
Protein metabolism									
Protein translation	1	0	2	1	9	0	0	0	0
Chaperonin-mediated protein folding	2	1	1	2	0	0	1	1	0
Different proteasome pathways	2	3	3	3	0	0	2	0	0
Endocytosis and traffic of neurotransmitter receptors									
Trafficking of AMPA receptors	6	1	1	0	0	0	0	1	0
Clathrin-mediated endocytosis	3	3	2	3	0	3	0	1	0
Total	18	17	13	12	9	6	7	6	5

### Intellectual disability and seizures are among the most common symptoms caused by metabolism proteins in the PSD

We first looked into the overall number of different phenotypes caused by these 60 diseases. Surprisingly, 836 different phenotypes were identified, indicating the complex nature of the conditions caused by these 53 proteins. This is further illustrated by the fact that most phenotypes are only found in one or two disorders (691/836, ≈ 83% of the total). Among the most frequent symptoms identified by HPO (Table 3), two are neurological: ‘Intellectual Disability’ (in 18 different diseases) and ‘Seizures’ (in 17). Other relevant neurological symptoms identified are ‘Microcephaly’, ‘Specific Learning Disabilities’, ‘Hyporeflexia’ or ‘Spasticity’. Some of these symptoms were much more commonly found in conditions caused by proteins from the same metabolic pathway. The most extreme case is that of ‘Spasticity’, which is only caused by proteins related to energetic metabolism. Another example is found in ‘Specific Learning Disabilities’ that is absent from diseases caused by the protein machinery involved in protein metabolism. Many of these diseases are related to the new categories of inborn errors of metabolism affecting systems involved in intracellular vesiculation, trafficking, processing of complex molecules and quality control processes (such as protein folding and autophagy) (García-Cazorla and Saudubray, this issue).

The elevated frequency of intellectual disability and seizures among disorders caused by proteins related to postsynaptic metabolic pathways is in favour of a postsynaptic role in some of these conditions. These two phenotypes are characteristic of disorders caused by genes coding for proteins with a very prominent role at the PSD. This is the case of ionotropic glutamate receptors and their auxiliary proteins (Soto et al. 2014; Bayés et al. 2014; Volk et al. 2015; Zehavi et al. 2017), *SYNGAP1* (Hamdan et al. 2009; Clement et al. 2012), *DLG3* (Tarpey et al. 2004), *SHANK2* (Berkel et al. 2010; Leblond et al. 2012), *SHANK3* (Guilmatre et al. 2014), *NEUROLIGIN 2* (Parente et al. 2017) or *OPHN1* (Billuart et al. 1998), to mention just a few. These phenotypic correspondences would support the notion that postsynaptic dysfunction might have a role in some neurometabolic disorders. Nevertheless, further research in this field must be done to corroborate this hypothesis.

## Conclusions

In the recently developed field of synaptopathies (Brose et al. 2010; Grant 2012), the role of many postsynaptic proteins in mental disorders is already well established (Guilmatre et al. 2009; Hamdan et al. 2011; Kirov et al. 2011). Nevertheless, the vast majority of postsynaptic proteins known to cause brain conditions are not involved in metabolism. In this article, I have explored the possibility of a primary role in disease of postsynaptic proteins involved in metabolic pathways. I have shown that energy production, protein turnover and neurotransmitter receptor traffic are the major metabolic pathways present at the postsynapse and that many proteins from these processes cause inherited disorders encompassing neurological manifestations. Importantly, the most common neurological phenotypes caused by these disorders are intellectual disability and seizures. Although, many other symptoms are present, including movement disorders, as recently reported (Kurian M, this issue). The fact that intellectual disability and seizures are key phenotypes of well-established synaptopathies caused by postsynaptic proteins and of conditions caused by postsynaptic proteins involved in metabolism is suggestive of a role of postsynaptic metabolism in certain neurological conditions. Future research will be required to explore this hypothesis.

## Electronic supplementary material

Supplementary Table 1 (XLSX 33 kb)

Supplementary Table 2 (XLSX 18 kb)
